# The Role of Biological Agents in the Management of Large Vessel Vasculitis (LVV): A Systematic Review and Meta-Analysis

**DOI:** 10.1371/journal.pone.0115026

**Published:** 2014-12-17

**Authors:** Mohammed Osman, Christian Pagnoux, Donna M. Dryden, Dale Storie, Elaine Yacyshyn

**Affiliations:** 1 Division of Rheumatology, Department of Medicine, University of Alberta, Edmonton, Alberta, Canada; 2 Division of Rheumatology, Department of Medicine, University of Toronto, Toronto, Ontario, Canada; 3 University of Alberta Health Sciences Library, Edmonton, Alberta, Canada; 4 Department of Pediatrics, University of Alberta, Edmonton, Alberta, Canada; University of Milan, Italy

## Abstract

**Background:**

Giant cell arteritis (GCA) and Takayasu's arteritis (TAA) are large vessel vasculitides (LVV) for which corticosteroids (CS) are the mainstay for treatment. In patients with LVV unable to tolerate CS, biological agents have been used with variable effectiveness.

**Objective:**

To systematically review the effectiveness and safety of biological agents in patients with LVV.

**Methods:**

We searched 5 electronic databases (inception to October 2012) and conference abstracts with no language restrictions. Two reviewers independently selected studies, extracted data and assessed methodological quality. Our protocol was registered in PROSPERO.

**Results:**

We included 25 studies (3 RCTs and 22 case series with ≥2 cases). 95 GCA and 98 TAA patients received biological agents. The RCTs using anti-TNF agents (infliximab, etanercept and adalimumab) did not suggest a benefit in GCA. GCA patients receiving tocilizumab, in case series, achieved remission (19 patients) and reduction of corticosteroid dose (mean difference, –16.55 mg/day (95% CI: –26.24, –6.86)). In case series, 75 patients with refractory TAA treated with infliximab discontinued CS 32% of the time. Remission was variably defined and the studies were clinically heterogeneous which precluded further analysis.

**Conclusion:**

This systematic review demonstrated a weak evidence base on which to assess the effectiveness of biological treatment in LVV. Evidence from RCTs suggests that anti-TNF agents are not effective for remission or reduction of CS use. Tocilizumab and infliximab may be effective in the management of LVV and refractory TAA, respectively, although the evidence comes from case series. Future analytical studies are needed to confirm these findings.

## Introduction

Large vessel vasculitis (LVV) includes two major forms, giant cell arteritis (GCA, temporal arteritis) and Takayasu's arteritis (TAA) [1]. Both diseases affect mostly females [2] and are defined by inflammatory changes within the walls of the aorta and/or its major branches [3]. GCA is the most common primary vasculitis in adults older than 50 years. Patients with GCA often present with symptoms stemming from ischemia corresponding to the affected arteries [4]. TAA, unlike GCA, affects females younger than 40 years [5]. Once the diagnosis of TAA is established and supported by vascular imaging, disease monitoring is difficult. Inflammatory markers are not always reliable and non-invasive vascular imaging techniques, including ^18^F-Fluorodeoxyglucose (^18^F-FDG) positron emission tomography (PET), computed tomography and magnetic resonance angiography (CTA and MRA, respectively) are still being optimized as tools to assess for disease activity [6].

The cause(s) and underlying mechanisms of inflammation in GCA and TAA are unknown. *Ex vivo* studies have suggested a role for self-reactive leukocytes producing mediators (TNF-α and IL6) which are thought to play a critical role in the pathogenesis of LVV [3]. Targeting some of these key players may be important in the management of both GCA and TAA.

Many patients with GCA or TAA initially respond to high doses (1 mg/kg/d) of corticosteroid (CS) therapy. However, they also remain on high doses for prolonged periods (1 to 2 years), and may develop long term serious sequelae and complications of CS use [7]. Furthermore, up to 60% of patients with LVV have relapses despite using CS for prolonged periods [8]. There are few therapeutic options for treating LVV beyond CS. Results of studies investigating disease modifying anti-rheumatic drugs (DMARDs, e.g. methotrexate) have been disappointing [9]. Anti-cytokine/immune cell depleting monoclonal antibodies/soluble receptors, or ‘biological agents' have been investigated as alternate agents to treat LVV; however, their effectiveness remains unclear [6], [10], [11].

The objective of this systematic review was to assess the effectiveness and safety of biological agents in the induction of remission for patients with GCA and TAA. Biological agents included anti-TNF-α agents (infliximab [IFX], adalimumab [ADA], etanercept [ETN]), anti-IL6R (tocilizumab [TCZ]), anti-CD20 (rituximab [RXB]), anti-IL-12/23 p40 (ustekinumab), and the soluble CTLA4 receptor fusion protein (abatacept). Our primary outcome was the establishment of disease remission in GCA or TAA patients; our secondary outcomes were the reduction of CS use after the addition of these agents, and their adverse effects.

## Methods

These methods are based on our protocol, which was registered in PROSPERO [12].

### Search Strategy

A research librarian (D. S.) conducted searches in MEDLINE (see **S1 Appendix** for the list of search terms), EMBASE, Cochrane Central Register of Controlled Trials (CENTRAL), Web of Knowledge, and Proquest Dissertations and Theses from inception to October 2012, without limitations for study design, age or language. The search strategy included different terms for GCA, TAA, LVV, and treatments with DMARDs and/or the following biological agents: IFX, ETN, ADA, TZB, RXB, ustekinumab, or abatacept. A hand search through the American College of Rheumatology (ACR) and European League Against Rheumatism (EULAR) databases for abstract proceedings (2009 to 2012) was also performed (M.O.). We contacted the corresponding and/or first authors of potentially relevant abstracts to obtain unpublished data and/or manuscripts. Abstracts that were subsequently published as journal articles were included as articles.

### Study selection, data extraction

We included randomized and nonrandomized controlled trials (RCTs and NRCTs) and observational studies (case-control, cohort studies and case series) if they included GCA or TAA patients receiving a biological agent. Single patient case reports or studies only having a single patient treated with a biological agent were excluded. We included abstracts from proceedings where the data were provided by authors.

Two reviewers (M. O., E. Y.) independently screened titles and abstracts using broad inclusion criteria. The full-text of all potentially relevant studies were assessed by the two reviewers independently using predefined eligibility criteria. Disagreements were resolved by consensus.

One reviewer (M.O.) extracted data using a standardized form; a second reviewer (E.Y.) verified data for accuracy and completeness. Information collected included patient demographics (number of patients, age, sex, disease duration), prior immunosuppressive therapies, biological agent(s) used, dose and frequency, inflammatory markers (erythrocyte sedimentation rate (ESR) and C-reactive protein (CRP)) before and after biological agent therapy, CS dose before and after therapy, disease remission (as defined by each study), number of patients able to stop CS, relapses, adverse effects, and follow-up periods. Because remission was defined using different parameters in each study, we highlighted studies that defined remission as normalization of clinical symptoms, using CS-equivalent of <10 mg per day of CS, normalization of inflammatory markers, and absence of new/active changes in follow-up radiography (MRA, CTA or PET/CT).

### Quality Assessment

The same two reviewers (M. O., E. Y.) independently assessed methodological quality of included studies. Disagreements were resolved by consensus. For RCTs, we used the Cochrane Risk of Bias tool to assess internal validity across seven domains (sequence generation, allocation concealment, blinding of participants and personnel, blinding of outcome assessment, incomplete outcome data, selective outcome reporting, and other sources of bias) [13]. We present an assessment of low, unclear or high risk of bias for each RCT. The overall assessment is based on the responses to individual domains. If one or more individual domains had a high risk of bias, we rated the overall score as high risk of bias. We rated the overall risk of bias as low only if all components are assessed as having a low risk of bias. In all other situations, the overall risk of bias was rated as unclear.

For case series, we used a checklist [14] that assessed consecutive enrolment, complete outcome data, and standardized/independent approach to outcome assessment. We present an assessment of low, unclear or high risk of bias for each case series.

### Data synthesis and analysis

Median values and range were calculated for disease duration, patient age, CRP and CS doses before and after receiving a biological agent, and median follow-up for each study and summarized in tables.

We performed meta-analyses using a DerSimonian and Laird random effects model after pooling the data from case series included when the population and biological agent were similar to assess the effectiveness of the agent in reducing CS dose [15]. Statistical heterogeneity was quantified using the I-squared (I^2^) statistic [16]. We calculated mean differences (MD) for continuous outcomes using the inverse variance method. For all estimates, we reported 95% confidence intervals (CIs). We used Review Manager, version 5.0 (The Cochrane Collaboration, Copenhagen, Denmark) to perform meta-analysis.

## Results

**Figure 1:**
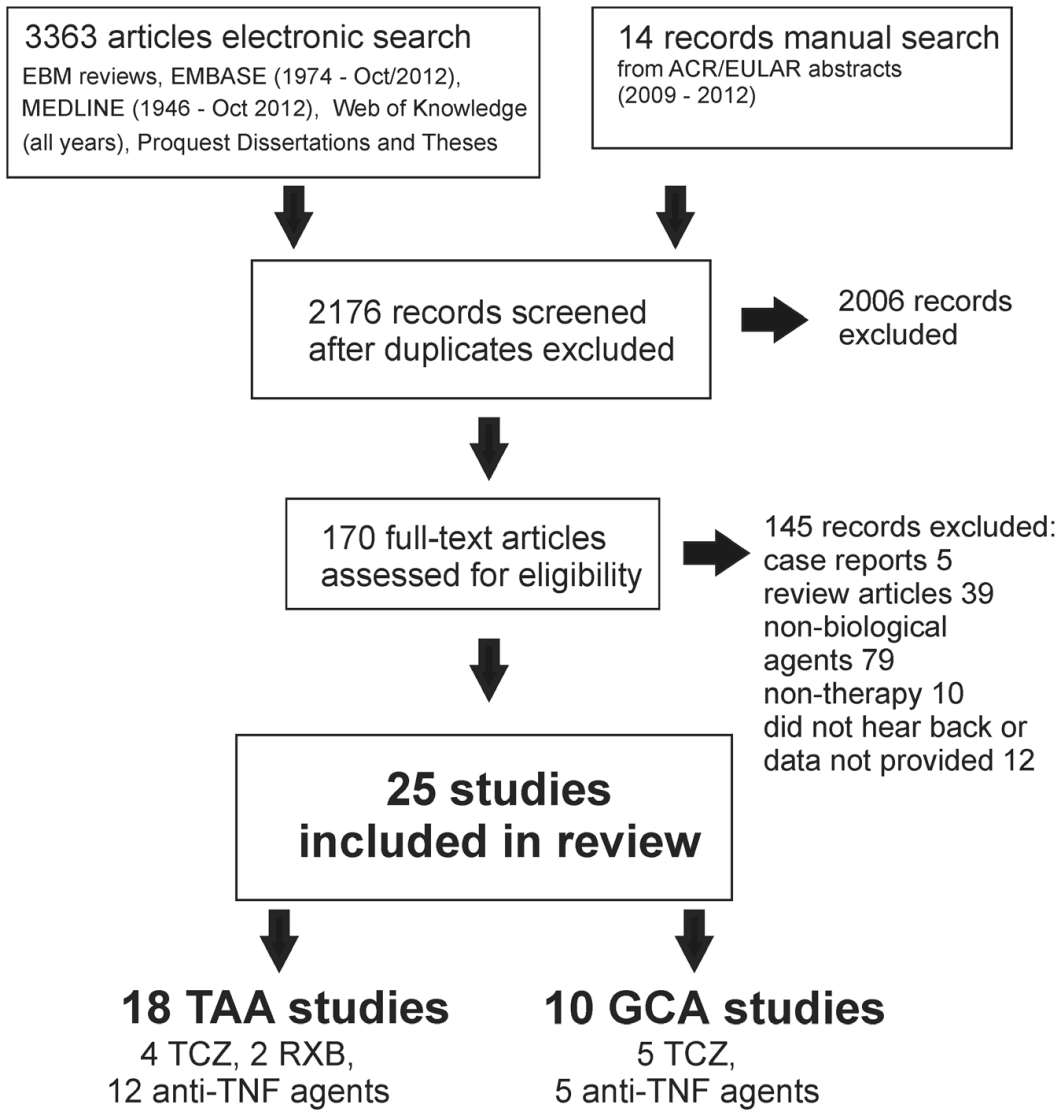
Schematic overview of the studies included in this review. 25 studies were included in this review. Of those, three studies included both GCA and TAA patients. One case series with TAA patients was not included in the analysis as its patients were included in a subsequent follow-up study [24], [42].

The electronic database and grey literature searches identified 3,377 citations (**Fig. 1**). Twenty-five studies met our inclusion criteria [17]–[40]. One study [38] had an associated publication that provided additional data [41]. Patients from one report were included in a subsequent follow-up study [24], [42]; we included patients only once in our analyses but used information from both studies. We included one abstract for which the authors provided sufficient data for inclusion [36].

We included 3 RCTs (n = 131 patients), and 22 case series (n = 150 patients) (**Tables 1** **–** **3**). Our search identified five studies in which their authors described them as cohort studies. However, because they were non-comparative in nature, we include them in our review as case series (**Table 2** [23]–[25], [40], [42]). One hundred and forty-five papers were excluded, mostly because they did not use biological agents (n = 79), were review articles (n = 39) or case reports (n = 5), or were non-therapy articles (n = 10). Of the fourteen abstracts we identified in our manual search, twelve authors did not respond or were not able to provide additional data. Only one study provided us with their unpublished data [36], while the other abstract was published, and thus, only the full length article was included in the analysis [18].

**Table 1:** Summary of randomized controlled trials for the use of biological agents in GCA.

Study and Primary endpoint	Biological agent	Number of patients		Median or Mean Age (years)		Female proportion (%)		Median Disease Duration		Remission rate		No. Pts. w/Prednisone <10 mg/d		Relapse rate		Follow-up Period	Risk of Bias	Generalizability
		PCB	Biological agent	PCB	Biological agent	PCB	Biological agent	PCB	Biological agent	PCB	Biological agent	PCB	Biological agent	PCB	Biological agent			
Hoffmann, GS *et. al,* 2007	IFX 5 mg/kg	16	28	69.5 (Median)	71.5	11/69 (69%)	24/28 (86%)	<4 weeks	<4 weeks	8/16 (50%)	12/28 (43%)	12/16 (75%)	17/28 (61%)	8/16 (50%)	16/28 (57%)	22 weeks	low	poor
																		
Relapse-free remission at 22 wks																		
																		
Martinez-Tapoada *et. al*, 2008	ETN 25 mg SC 2x/week	9	8	74.4 (Mean)	74.5	8/9 (88%)	7/8 (75%)	8.3 mo (1–53)	9.9 mo (2.7–24.9)	2/9 (22%)	4/8 (50%)	2/9 (22%)	4/8 (50%)	1/1	1/4 (25%)	15 months	high	poor
																		
steroid-free remission after 12 months																		
																		
Seror, R *et. al*, 2013	ADA 40 mg SC q2 weeks	36	34 (only 27 pts Rx)	74.5 (Median)	74.5	28/36 (77.8%)	24/34 (70.6%)	<2 weeks	<2 weeks	18/36 (50%)	20/34 (58.9%)	18/36 (50%)	20/34 (58.9%)	26/35 (74.3%)	20/27 (74.1%)	52 weeks	low	poor
																		
Remission at 26 weeks with CS <0.1 mg/kg																		
																		

The total number of patients enrolled before randomization was used for the analysis of each study's primary end point. Abbreviations: IFX infliximab), ADA (adalimumab), ETN (etanercept), SC (subcutaneously), PCB (placebo (CS alone).

**Table 2:** Summary of case series using biological agents in GCA.

Study	Biological Agent	No. of patients	Median age (range (y))	Female proportion	Median disease duration (mo, (range))	Remission rate	Median CRP		Median Prednisone (mg/d)		Pts w/o Prednisone in remission	relapse rate	Median follow-up period	Overrall Quality***
							before	after	before	after				
Beyer *et. al*, 2011*, **	TCZ	3	72 (71–79)	2/3	NR	3/3	34.9	<4	30	<7.5	0/3	0/3	6	good (−/+/+)
Sciascia *et. al,*2011	TCZ	2	76.5 (76–77)	2/2	NR	2/2	NR	NR	25	5	0/2	0/2	7	fair (−/+/−)
Seitz *et. al*, 2011 *	TCZ (2 monoRx)	5	71 ((63–79)	3/5	6 (3–56)	5/5	14	<3	20	5	3/5	0/5	8.3	good (+/+/+)
Salvarani *et. al*, 2012 *, **	TCZ (1 monoRx)	2	59 (54–64)	0/2	19 (2, 36)	2/2	51	0.4	12.5	1.25	1/2	1/2	9.5	good (−/+/+)
Unizony *et. al*, 2012 **	TCZ	7	69 (mean, 60–83)	NR	NR (10–24)	7/7	34	0.7	15	0.5	4/7	2/7	7	fair (−/+/−)
Cantini *et. al*, 2001 **	IFX	4	74 (72–75)	3/4	47.5 (42–54)	3/4	46	3	12.5	0	3/4	0/3	5	fair (−/+/−)
Andonopoulos *et. al*, 2003	IFX	2	82.5 (80–85)	0/2	NR	2/2	51	0.4	0	0	1/2	2/2	4.5	fair (−/+/−)

Three studies (Seitz *et. al*, 2011 [19], Salvarani *et. al*, 2012 [21] and Unizony *et. al*, 2012 [17]) also appear in Table 3 with TAA patients. Abbreviations: TCZ (tocilizumab), monoRx (monotherapy), IFX (infliximab), CRP (C-reactive protein), NR (not reported), N/A (not applicable), F/U (followup).

* In these studies, remission was defined using clinical, biochemical and the absence of new radiographic findings.

** In these studies, F/U was> 6 mo while patients were in remission.

*** Quality Assessment was conducted as follows: Cases series: pts enrolled consecutively or randomly vs hand-picked? Did> 90% of the pts enrolled get analyzed? Was a standardized approach used to assess remission (clinical/biochemical/radiographic). Study quality also reflects risk of bias.

**Table 3:** Summary of case series using biological agents in TAA.

Study	Biological Agent	No. of patients	Median age (range (y))	Female proportion	Median disease duration (mo, (range))	Remission rate	Median CRP		Median Prednisone (mg/d)		Pts w/o Prednisone in remission	relapse rate	Median follow-up period	Overrall Quality***	
							before	after	before	after					
Seitz *et. al*, 2011 *	TCZ	2	33.5 (27–40)	2/2	57 (42–72)	1/2	45.5	3	35	2.5	0/2	1/1	6	good (+/+/+)	
Salvarani *et. al*, 2012 *, **	TCZ (1 monoRx)	2	30.5 (21–40)	2/2	7.5 (3, 12)	2/2	40.2	8.5	12.5	0	2/2	0/2	8.5	good (−/+/+)	
Unizony *et. al*, 2012	TCZ	2	41.5	2/2	NR	2/2	12.3	2.95	5	0	2-Feb	0/2	9.5	fair (−/+/−)	
Canas *et. al, 2012* **	TCZ	5	30 (12–32)	3/3	48 (3–96)	3/3	26.1	1	NR (<10)	NR (<10)	0/2	0/5	6	fair (−/+/−)	
Galarza *et. Al, 2008*	RXB	2	27 (27–29)	2/2	NR	2/2 (not clear)	NR	NR	NR	NR	NR	NR	NR	poor (−/?/−)	
Hoyer *et. al*, 2012*	RXB	3	18 (12–31)	3/3	48 (48–120)	3/3	90	5.0	7.5	7.5	0/3	NR	NR	fair (−/?/+)	
Della Rosa *et. al*, 2005 **	IFX	2	19.5 (16–23)	2/2	35 (35–40)	2/2	68.5	normal (NR)	8	2	1/2	0/2	8.5	fair (−/+/−)	
Hoffman *et. al*, 2004*, **, #	IFX	8/15	28.5 (17–48)	14/15	60 (15–110)	7/8	NR	NR	20	2.5	3/8	5/8	11 (3–40)	good (−/+/+)	
	ETN to IFX	2/15	23.5 (19–28)		15 (12–18)	2/2	NR	NR	22.5	0	2/2	2/2	32.5 (17–48)		
	ETN	5/15	25 (19–42)		30 (2–160)	5/5	NR	NR	20	0	5/5	5/5	28 (11–35)		
Molloy *et. al*, 2008*, **, #	IFX	21/25	35 (15–64)	22/25	116 (39–344)	23/21	NR	NR	19 (5–50)	0 (0–30)	12/21	2/12	28 (4–82)	good (−/+/+)	
	ETN	4/25	mean			3/4	NR	NR			2/3	2/4			
Karageorgaki *et. al, 2007* *, **	IFX	4	25 (17–32)	4/4	57 (24–86)	2/4	54	16	5.63	8.75	0/4	1/2	14	fair (−/−/+)	
Filacomo *et. al*, 2008 **	IFX	4	11.5 (7–12)	4/4	10.5 (1–30)	2/4	NR	NR	not clear	5	0/2	2/4	6	good (+/+/−)	
Nune *et. al, 2010* *, **	IFX	3/15 Rx IFX	21 (17–21)	3/3	48 (24–180)	3/3 (no repeat imaging)	NR	NR	20	10	1/3	NR	6–10	good (+/+/−)	
Kaneko *et. Al, 2010* **	IFX	3	17 (16–17)	3/3	6 (3–11)	2/3	NR	NR	20	0 (1 pt pulsed)	2/3	1/2	6	fair (−/+/−)	
Buonomo *et. al*, 2011**	IFX	2	15 (14–16)	3/3	6 (3–11)	2/3	NR	NR	0.5 mg/kg	NR (lower)	0/2	0/2	NR	fair (−/+/−)	
Osman *et. al*, 2011	IFX then ADA	2	28 (17–39)	2/2	28	0/2	NR	NR	40	25	N/A	N/A	NR	poor (−/−/−)	
Commarmond *et. al*, 2012**	IFX	2	26 (24–28)	0/2	21 (6–36)	2/2	59	<4	22.5	5	0/2	0/2	101.5	fair (−/−/+)	
Mekinian e*t. al*, 2012**	IFX	15	41 (17–61)	13/15	36 (6–365)	11/15	30	9	20	6	1/15	4/11	43	fair (−/+/−)	
Schmidt et. Al, 2012*. **	IFX	17/20	29.8 (30–44)	19/20	15.9 (2–32.7)	18/20	10	NR	12.2 (3–25)	NR	7/12	6/18	54 (34–82)	good (−/+/+)	
	ADA	2/20													
	ETN	1/20													

Abbreviations: TCZ (tocilizumab), monoRx (monotherapy), IFX (infliximab), CRP (C-reactive protein), NR (not reported), N/A (not applicable), F/U (followup), RXB (rituximab), ETN (etanercept), ADA (adalimumab).

* In these studies, remission was defined using clinical, biochemical and the absence of new radiographic findings.

** In these studies, F/U was> 6 mo while patients were in remission.

*** Quality Assessment was conducted as follows: Cases series: pts enrolled consecutively or randomly vs hand-picked? Did>90% of the pts enrolled get analyzed? Was a standardized approach used to assess remission (clinical/biochemical/radiographic). Study quality also reflects risk of bias.

# These two studies were from the same cohort and the latter was a follow-up of the initial one. The patients were only analysed once, but quality assessments were conducted for both studies.

Patients in the RCTs were treated with three different biological agents: IFX [30], ETN [26] and ADA [18]. In the case series, the biological agents included: IFX (n = 14 [22]–[25], [27], [28], [32]–[35], [37], [39], [40], [42]]); ETN (n = 3 [24], [40], [42]; ADA (n = 2 [22], [40]); RXB (n = 2 [29], [31]), and TCZ (n = 6 [17], [19]–[21], [36], [38]). None of the studies used abatacept or ustekinumab.

For the most part, the studies appropriately classified their patients with LVV (GCA or TAA). One case series classified three of their patients as having TAA, but we excluded them from the analysis as these patients were age>50 years at diagnosis [34].

### Quality Assessment

Two RCTs were assessed as low risk of bias [18], [30]; however, they had poor applicability to patients with GCA as their steroid tapering regimens were too rapid and not reflective of how GCA patients often require a more prolonged course of CS [18], [30]. One RCT had a high risk of bias due to selection bias (both random sequence generation and allocation concealment) and attrition bias; detection bias was unclear [26]. All three RCTs received funding or support from pharmaceutical companies.

Of the 22 case-series, we rated 8 as good quality [19], [21], [23], [24], [32], [38], [40], [42], 12 as fair quality [17], [20], [25], [27]–[29], [33]–[37], [39], and 2 with poor quality [22], [31] (**Tables 1** **and** **2**).

### Patient Demographics

The 25 studies included 193 patients treated with a biological agent – 95 GCA patients and 98 TAA patients. An equivalent proportion of GCA patients treated with a biological agent were treated with IFX and ADA (35.6%), followed by TCZ (20%), and ETN (8.4%). Most of the TAA patients were treated with IFX (76.5%), followed by TCZ (11.2%), ETN, RXB (both 5.1%), and finally ADA (2.0%).

As expected, most of the GCA patients treated with a biological agent were female (65 patients, 73.9%). Patient ages ranged from 58 to 85 years (**Tables 1** **and** **2**). The median disease duration was variable. Patients in the ADA and IFX RCTs were newly diagnosed with GCA (<2 and 4 weeks from diagnosis, respectively) [18], [30], whereas patients in the ETN trial had a median duration of 9.9 months [26]. TCZ patients from these studies typically had a variable duration (2–56 months).

A majority of the TAA patients were female (89.7%) (**Table 3**). These patients were young with a median age of 28–30 years (range 7–48) (**Table 3**). Patients typically had long disease durations prior to the initiation of treatment with the biological agent (median 36 mo, IQR 70 mo). Methotrexate was the most commonly used DMARD, although other agents had also been used.

### Effectiveness

#### Infliximab

In the RCT [30] using IFX for GCA patients (**Table 1**), the authors included newly diagnosed GCA patients (<4 weeks) who responded to CS prior to randomization, and compared IFX (5 mg/kg) and CS to placebo (CS alone) using 2∶1 randomization (21 vs. 16 patients). CS were rapidly tapered using a pre-specified regimen. The authors determined the number of patients that remained relapse free at 22 weeks and the adverse effects. There was no difference in the number of GCA patients who relapsed (43% vs. 50%, respectively, P = 0.65, RR 0.86 (95% CI, 0.45–1.65) or had a reduction in their CS doses to 10 mg/d (61% vs. 75%, P = 0.31, RR 0.81 (95% CI, 0.54–1.22).

Two case series (6 patients) presented GCA patients with both newly diagnosed and long-standing disease treated with IFX [35], [39]; follow-up was between 2 and 6 months. Both groups showed a modest benefit for IFX as five (83%) achieved remission and a reduction of CS doses and none developed a relapse during follow-up (**Table 2**). One of these studies [39] (n = 2) reported on newly diagnosed patients with GCA and treated with IFX without CS. Both patients had an initial response to IFX monotherapy, but relapsed within the follow-up period of 3 months (**Table 2**).

In 11 case series of 75 TAA patients treated with IFX, 74.7% (56/75 patients) achieved remission and 32% discontinued CS therapy during follow-up (**Table 3**). Of the patients that achieved remission, 28.6% (16/56 patients) developed a relapse; some studies did not report the frequency of relapses (**Table 3**). Reduction of CS dose results from the case series were not pooled because they were too clinically heterogeneous (**Table 3**). The follow-up periods in the case series ranged from 6 to 101 months.

#### Tocilizumab

**Figure 2:**
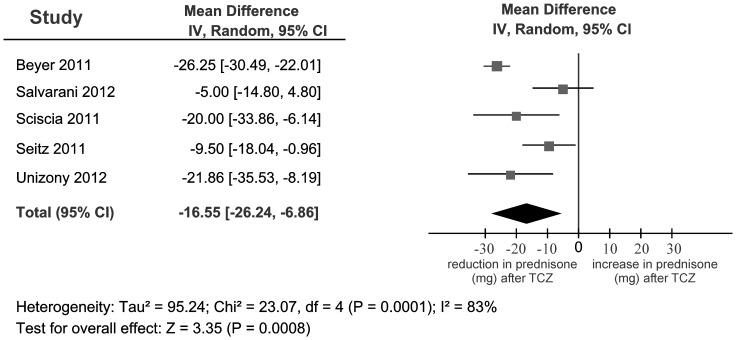
Meta-analysis plot of prednisone mean differences (mg) for GCA patients treated with TCZ from case series.

Five case series of patients with GCA reported on TCZ; follow-up periods ranged from 3 to 12 months. Of the 19 GCA patients treated with TCZ plus prednisone, all achieved disease remission (**Table 2**) and a reduction of CS doses (pooled mean dose reduction of 16.55 mg per day; 95% CI −26.24, −6.86; I^2^ = 83%) (**Fig. 2**). Although our meta-analysis showed substantial heterogeneity, we cannot attribute the heterogeneity to study design, disease duration, patient demographics, temporal artery positivity, or the use of TCZ monotherapy. Three GCA patients were treated with TCZ and no CS (**Table 2**) while nine completely discontinued CS by the end of the follow-up periods. Three (16%) patients treated with TCZ developed a relapse during the follow-up period.

Four case series reported on 11 patients with TAA who received TCZ; follow-up ranged from 3 to 12 months. Most (91%) achieved remission including one with TCZ monotherapy. All patients had a reduction of CS use (**Table 3**), with four being CS-free (36%), and many discontinuing their other immunosuppressive medications. Two patients that achieved remission relapsed during the follow-up period.

#### Adalimumab

One RCT [18] compared ADA plus CS with CS alone in GCA patients (**Table 1**, mean age 74.5 years). The authors enrolled patients with newly diagnosed GCA who had received CS for less than 2 weeks prior to randomization. Patients were randomized (1∶1) to receive ADA (40 mg sc every 2 weeks for 10 weeks; 34 patients) and CS or CS alone (placebo; 36 patients). The CS doses were quickly tapered using a predetermined regimen. The authors determined the number of patients that had <0.1 mg/kg of CS at 26 weeks in remission, their primary outcome, while secondary outcomes included the difference in CS use between both groups at 6 months, the ratio of relapse-free patients, and safety of ADA. ADA was not effective in maintaining remission in newly diagnosed GCA patients compared to placebo (58.9% vs. 50%, respectively, P = 0.46, RR 1.20 (95% CI [0.733 to 1.974])). It also did not reduce the amount of CS (0.12 mg/kg/day vs. 0.13 mg/kg/day, respectively, P = 0.71) or the relapse rate at 26 weeks (74.1% vs. 74.3%, respectively, **Table 1**). The authors observed this may have stemmed from the rapid CS tapering regimen that was employed [18].

#### Etanercept

In the RCT comparing ETN and placebo, ETN was not beneficial for patients with GCA [26]. Patients with longstanding biopsy-proven GCA with CS-induced adverse effects were randomized to receive either ETN (25 mg subcutaneously two times a week; 8 patients) or placebo (9 patients) after 12 months of follow-up. Four out of eight patients treated with ETN were able to control their disease with a reduced CS dose, however, the difference was not statistically significant (50% vs. 22%, respectively, P = 0.03, RR 1.83 (95% CI [0.698 to 4.812)) respectively)) [26], and only 4 patients treated with ETN were followed to the completion of the study (**Table 1**).

ETN was also not effective in maintaining remission in TAA. In two case series, 4 TAA patients receiving ETN, remission and reduction of CS doses were achieved in 3 patients, but 2 of them relapsed within the follow-up periods of each study (**Table 3**) [24], [42]. Two patients initially treated with ETN were not controlled on ETN monotherapy and were switched to IFX in order to control their disease (**Table 3**) [42]. The follow-up period for TAA patients treated with ETN was 4 to 82 months.

#### Rituximab

Two case series examined RXB for TAA patient (n = 5). One study did not provide information on how they defined remission, the CS doses, or other objective findings for disease remission [31]. The other study showed all of the patients treated with RXB (n = 3) achieved remission, but no patients had a reduction in CS use [29].

### Adverse Effects

The adverse effects for each biological agent are summarized in **Table 4**. Of note, 5/19 (26.3%) patients treated with TCZ were reported to have a transient, self-limited transaminitis [17], [21]. Some patients also developed leucopenia; however, they did not have increased infections. One patient developed a post-operative myocardial infarction, and autopsy demonstrated active GCA despite normal clinical, serological and radiographic values [17].

**Table 4:** Summary of adverse effects in GCA/TAA pts treated with biological agents.

Type of LVV	Biological agent	Number of patients	Number of Studies	Number pts. (%) with AE	Cessation rate*	Adverse Effects	
						Infections	Miscellaneous effects
**GCA**	TCZ	19	5	11 (36.8%) **	0	0	4 leukopenia, 5 transaminitis, 1 adrenal insufficiency and 1 post-op MI resulting in death
							
							
	IFX	33	3	26 (78.9%)	3/33 (12.2%)	20/33 (60.6 %)	1 heart failure, 6 infusion reactions
							
							
	ETN	8	1	8 (100 %)	3/8 (37.5 %)	4/8 (50 %)	1 heart failure, 1 N/V/weight loss, 2 transaminitis, 1 injection reaction
							
							
	ADA	34	1	24 (70.59)	5/34 (14.7)	16/34 (47 %)	1 injection site reaction, 1 breast CA
							
							
**TAA**	TCZ	11	4	0	N/A	N/A	N/A
							
							
	IFX	85	12	23/85 (27%)	11/85 (15.2%)	11/23 (47.8%)	steroid psychosis, breast CA transaminitis, allergic rash, allergy, serum sickness, pancreatic CA (all 1 pt) 4 infusion reactions
							
							
	ETN	12	3	3/12 (25%)	1/4	2/4	N and HTN (1 pt in total)
							
							
	ADA	3	2	NR	NR	NR	NR
							
							
	RXB	5	2	NR	NR	NR	NR
							
							

Abbreviations: A/E (adverse effects), TCZ (tocilizumab), IFX (infliximab), ETN (etanercept), ADA (adalimumab), RXB (rituximab), N (nausea), V (vomiting), N/A (not applicable), NR (not reported), HTN (hypertension), MI (myocardial infarction).

* Cessation rate - discontinuation secondary to adverse effects;

** Likely an overestimation as one study did not specify which patients developed leukopenia and transaminitis

IFX was associated with more adverse effects, particularly infections and infusion reactions some of which resulted in cessation of treatment (**Table 4**). One GCA patient developed clinically significant heart failure in the IFX RCT [30]. Two TAA patients from case series developed malignancies – breast and pancreatic cancers. The patient who developed pancreatic adenocarcinoma was previously on azathioprine, possibly an underlying risk [40]. Patients treated with ETN had higher rates of infection; one patient developed heart failure [26], [42]. For GCA patients there was no difference in the rate of general and serious adverse effects for those treated with ADA compared to placebo from RCT data [18].

## Discussion

This systematic review identified 25 studies (3 RCTs, 22 case series) of LVV patients treated with five biological agents: IFX, TCZ, ETN, ADA and RXB. The results of the RCTs show that TNF agents are not effective in inducing remission or in reducing CS doses in patients with GCA. On the other hand, results from case series of patients with GCA and TAA suggested that TCZ may be of some benefit for the maintenance of remission, and for the reduction of CS use. Case series results also suggested that IFX may be beneficial in the maintenance of remission and possibly reducing the amount of CS use in TAA patients. Our review also suggests that TCZ may be a safe alternative for patients with GCA and TAA, although our data stems from case series and the follow-up periods were not very long. IFX may be associated with increased risk of complications such as infections in both GCA patients and TAA patients.

Our review included GCA patients in case series that were relatively comparable in their disease duration to TAA patients (median 36 mo, IQR 44 mo (6–50) and 36 mo, IQR 70 mo (15–85); respectively) which likely reflects the selection bias inherent to patients with refractory GCA and TAA that have failed multiple previous therapies. In fact, many patients with both GCA and TAA had failed other CS-sparing therapies prior to switching to a biological agent, although one study included patients that were treated with TCZ monotherapy [21]. From our review, it does not appear that ADA, RXB or ETN are effective for inducing and/or maintaining clinical remission, as defined by clinical, biochemical and/or radiographic parameters and a reduction of CS to <10 mg/d, for both GCA and TAA. No studies reported data on abatacept or ustekinumab in GCA or TAA.

All of the evidence supporting the use of biological agents for LVV comes from case series. In contrast to the results of the RCTs, many of the case series for GCA showed some benefit for the use of TNF agents. This may reflect the underlying bias present in observational studies such as case series. It may also reflect differences in study populations and drug protocols. For example, patients treated with IFX in case series had already been treated for prolonged periods with CS prior to IFX therapy [35] while those treated in the RCT using IFX were newly diagnosed and were treated with CS using a predetermined rapid tapering regimen [30]. In addition, many TAA patients maintained disease remission by IFX dose escalation [24]. The differences in response to IFX between GCA and TAA may also represent differences between the pathogenesis of cranial GCA and systemic TAA. Subsequent RCTs using TCZ in GCA or IFX in TAA, for example, may not show a benefit for similar reasons. Future studies evaluating a role for TCZ in GCA may suggest a possible benefit as authors of ongoing studies [43] have recognized and tried to address external validity concerns present in previous RCTs [18], [30] such as treating patients LVV with a pre-specified short course of CS. Given the inherent weaknesses of case series in their study design and the high risk for publication bias, these results must be interpreted with caution.

An inherent problem with studying LVV and its management is the variable definition of disease remission. Many studies defined LVV remission as the absence of symptoms, and normalization of inflammatory markers (ESR and CRP). However, inflammatory markers are not completely reliable [44] – especially when TCZ inhibits IL-6 from binding to its receptor and IL-6 is required for CRP synthesis from the liver [45]. This is highlighted in one of the studies using TCZ where one patient was noted to be in remission; however, autopsy results suggested active disease [17]. Moreover in TAA, remission should not solely be monitored using inflammatory markers or clinical outcomes as these parameters may not reflect radiographic progression [46], [47]. In a minority of the studies (9 out of 22), remission was defined using the combination of clinical parameters, inflammatory markers, and the absence of new radiographic changes suggesting disease activity during the follow-up periods. PET-CT, unlike other imaging modalities, has been suggested to identify pre-stenotic lesions in TAA patients, and may be useful in monitoring patients with relapses [48]. Future analytical (e.g. RCTs and prospective cohort studies) studies using accepted criteria for remission in TAA are required both for agents showing some benefit in our review (TCZ and IFX) and ones with little or no evidence (e.g. RXB, ADA). Also, many questions still remain: addressing the frequency of relapses in longer follow-up of TCZ patients; validating inflammatory biomarkers for LVV patients treated with TCZ, the utility of traditional DMARDs (e.g. MTX) in LVV patients; and weighing the cost/benefit of biological agents with their efficacy and adverse effects. Only well-designed trials will begin to answer these pertinent questions.

In summary, after reviewing the existing literature to assess the role of biological agents in inducing and maintaining remission in LVV patients, only a small number of studies met our inclusion criteria. Although we showed a potential benefit for TCZ in both GCA and TAA, and IFX in TAA for both disease remission and for CS-sparing, all the evidence comes from small case series, which suffer from many biases and limitations. There is a paucity of RCTs at this time evaluating a role for biological agents in LVV. Our systematic review is the most up to date critical and comprehensive review in this area. Unlike other studies, our systematic review is the first to use a study protocol published *a priori*, includes a gray-literature search and directly evaluates the level of bias and the validity/interpretation of results from each included study. Well-designed studies are desperately needed in order to increase our understanding of the potential role of biological agents in the management of LVV.

## Supporting Information

S1 Appendix **Search Strategy for included articles.**
(DOCX)

S1 Checklist **PRISMA Checklist.**
(DOCX)
