# Supplementary material for: The Role of Biological Agents in the Management of Large Vessel Vasculitis (LVV): A Systematic Review and Meta-Analysis
Source: PLoS One. 2014 Dec 17;9(12):e115026. doi: 10.1371/journal.pone.0115026 (PMC4269410; doi:10.1371/journal.pone.0115026)
Supplement: S1 Appendix — Search Strategy for included articles. (DOCX) [file pone.0115026.s001.docx]

**Appendix S1. Search Strategy for included articles.**

**Searches and Databases**

No restrictions on language, or publication period.

With the assistance of a medical librarian, the following databases will be searched: Medline, EMBase, Proquest (for theses) and the Cochrane Library (EBM reviews). A manual search of abstracts presented at the annual meetings of the European League Against Rheumatism (EULAR), American College of Rheumatology (ACR), and clinicaltrials.gov from 2009 to 2012 was performed.

Search strategy:

**Medline** (Ovid 1946 – present)

MeSH terms: (vasculit* or arterit*) and large vessel).mp; (giant cell or temporal or cranial or granulomatous) adj1 (arterit** or aortiti*)) or (horton* adj1 disease*)).mp; (takayasu* or aortitis).mp; takayasu arteritis/dt; aortitis/dt; infliximab.mp; adalimumab.mp; etanercept.mp; tocilizumab.mp; methotrexate.mp; azathioprine.mp; exp Mycophenolic Acid/ or mycophenolate mofetil.mp; rituximab.mp; remicade.mp.; humira.mp; enbrel.mp; actemra or roactemra).mp; (Abitrexate or Folex or Mexate or Rheumatrex or Trexall or Metoject or Rheumatrex or Ebetrex).mp; (Azasan or Imuran).mp; (cellcept or myfortic).mp; (rituxan or mabthera).mp; exp Antibodies, Monoclonal/exp Antirheumatic Agents; leflunomide.mp.; arava.mp; Isoxazoles; exp Cyclophosphamide/cyclophosphamide.mp; Cytoxan or Neosar or Endoxan or Procytox).mp; abatacept.mp; orencia.mp; ustekinumab.mp; stelara.mp; Tumor Necrosis Factor-alpha/ai; exp anti-inflammatory agents, non-steroidal; exp CTLA-4 Antigen/ai [Antagonists & Inhibitors]; ((tnf alpha or tumor necrosis factor alpha) adj2 (inhibitor* or anti)).mp; exp Immunosuppressive Agents.

**EMBASE** (1974 – present)

MeSH terms: (vasculit* or arterit*) and large vessel).mp; (((giant cell or temporal or cranial or granulomatous) adj1 (arterit** or aortiti*)) or (horton* adj1 disease*)).mp; (takayasu* or aortitis).mp; infliximab.mp.; adalimumab.mp; etanercept.mp; tocilizumab.mp; methotrexate.mp; azathioprine.mp; exp Mycophenolic Acid/ or mycophenolate mofetil.mp; rituximab.mp; remicade.mp; humira.mp; enbrel.mp; (actemra or roactemra).mp; (Abitrexate or Folex or Mexate or Rheumatrex or Trexall or Metoject or Rheumatrex or Ebetrex).mp; (Azasan or Imuran).mp.; (cellcept or myfortic).mp.; (rituxan or mabthera).mp; leflunomide.mp; arava.mp; Isoxazoles/exp; Cyclophosphamide/cyclophosphamide.mp; (Cytoxan or Neosar or Endoxan or Procytox).mp; abatacept.mp; orencia.mp; ustekinumab.mp; stelara.mp; ((tnf alpha or tumo?r necrosis factor alpha) adj2 (inhibitor* or anti)).mp; exp *monoclonal antibody.

**EBM Reviews - includes the following databases:**

EBM Reviews - ACP Journal Club 1991 to October 2012; EBM Reviews - Cochrane Central Register of Controlled Trials October 2012; EBM Reviews - Cochrane Database of Systematic Reviews 2005 to September 2012; EBM Reviews - Database of Abstracts of Reviews of Effects 3rd Quarter 2012; EBM Reviews - Cochrane Methodology Register 3rd Quarter 2012; EBM Reviews - Health Technology Assessment 4th Quarter 2012; EBM Reviews - NHS Economic Evaluation Database 4th Quarter 2012.

MeSH terms: ((vasculit* or arterit*) and large vessel).mp; (((giant cell or temporal or cranial or granulomatous) adj1 (arterit* or aortiti*)) or (horton* adj1 disease*)).mp; (takayasu* or aortitis).mp.

**Web of Knowledge** - (includes Science Citation Index and Science Conference Proceedings)
Topic=("giant cell arteritis" OR "large vessel vasculitis" OR takayasu OR "temporal arteritis" OR "horton disease").

AND
Topic=(infliximab or adalimumab or etanercept or tocilizumab or methotrexate or azathioprine or mycophenol* or rituximab or remicade or humira or enbrel or actemra or roactemra) OR Topic=(abitrexate or folex or mexate or rheumatrex or trexall or metoject or rheumatrex or ebetrex or azasan or imuran or cellcept or myfortic or rituxan or mabthera or leflunomide or arava or isoxazoles or cyclophosphamide or cytoxan or neosar or endoxan or procytox or abatacept or orencia or ustekinumab or stelara or "tnf alpha inhibitor" or "tnf alpha antagonist" or "anti tnf" or "anti tumor necrosis factor" or "tumor necrosis factor alpha inhibitor" or "tumor necrosis factor alpha antagonist").

**Types of studies included and excluded**

Included: case-control studies, randomized control studies (both blinded and non-blinded), observational cohort studies, case series with all GCA and/or TA patients receiving the described biological /disease modifying immunsuppressive agents for the induction/maintenance of remission. The reason why disease modifying agents are included in the search is to capture patients treated with these agents and biological agents concomitantly.

Excluded: case reports and all case-control studies, randomized control studies, observational cohort studies, case series not using disease remission or maintenance of disease remission as a primary outcome, and studies using non-steroidal immunosuppressive agents without any biological agents.
